# Items

**Published:** 1880-02

**Authors:** 


### Items.

OFFICIAL LIST OF CHANGES OF STATIONS AND DUTIES of MEDICAL OFFICERS OF THE UNITED STATES MARINE HOSPITAL SERVICE. *October* 1, 1879, *to December* 31, 1879.

*Bailhache*, P. H., surgeon, relieved from duty, port of New York, and ordered to Philadelphia, temporarily. Thence to resume his duties as member of the National Board of Health, Oct. 25, 1879.

*Hutton*, W. H. H., surgeon, relieved from duty, port of New Orleans, and ordered to Detroit. Nov 1, 1879.

*Fessenden*, C. S. D., surgeon, relieved from duty, port of Portland, Maine, and ordered to New York. Oct. 25, 1879.

*Doering*, E. J., surgeon, relieved from duty, port of Philadelphia, and ordered to Portland, Maine. Oct. 25, 1879.

*Austin*, H. W., assistant surgeon, relieved from duty, port of Key West, and ordered to New Orleans. Nov. 1, 1879.

*Stoner*, G. W., assistant surgeon, relieved from duty, port of Buffalo, and ordered to Philadelphia. Oct. 25, 1879.

*Fisher*, J. C., assistant surgeon, detailed as chairman of board for the physical examination of candidates for promotion in the revenue marine service, Nov. 20, 1879. Granted leave of absence for nine days from Dec. 19, 1879. Dec. 17, 1879.

*Godfrey*, John, assistant surgeon, ordered to proceed to New Orleans for temporary duty during illness of surgeon Austin. Dec. 29, 1879.

*Goldsborough*, C. B., assistant surgeon, detailed as recorder of board for the physical examination of candidates for promotion in the revenue marine service. Nov. 20, 1879.

*White*, R., jr., assistant surgeon, ordered to Buffalo, Oct. 24,

1879. Orders to Buffalo revoked; ordered to report to surgeon Fessenden, New York, for duty. Nov. 12, 1879.

*Irwin*, Fairfax, assistant surgeon, upon expiration of leave of absence to proceed to Charleston, S. C. Oct. 31, 1879.

*Glazier*, W. C. W., assistant surgeon, relieved from duty, port of Charleston, S. C., and ordered to Key West, Fla. Oct. 31, 1879.

*Cooke*, H. B., assistant surgeon, relieved from duty, port of Pensacola, Fla., and ordered to Buffalo, N. Y. Dec. 1, 1879.

*O'Connor*, F. J., assistant surgeon, ordered to Philadelphia for temporary duty, Nov. 10, 1879. Ordered to Buffalo, N. Y., for temporary duty, Nov. 12, 1879. When relieved by assistant surgeon Cooke, to rejoin his proper station. Dec. 9, 1879.

*Porter*, F. D., assistant surgeon, assigned to temporary charge at Detroit Marine Hospital, Oct. 25, 1879. When relieved by surgeon Hutton, to proceed to Chicago and report for duty to Surgeon Miller. Dec. 17, 1879.

PROMOTIONS.

*Austin*, H. W., surgeon, promoted to be surgeon from Nov. 1, 1879. Nov. 3, 1879.

*Gassaway*, J. M., passed assistant surgeon, promoted to be passed assistant surgeon. Dec. 12, 1879.

*Smith*, Henry, passed assistant surgeon, promoted to be passed assistant surgeon. Dec. 12, 1879.

*Stoner*, G. W., passed assistant surgeon, promoted to be passed assistant surgeon. Dec 12, 1879.

RESIGNATIONS.

*Ellinwood*, C. N., surgeon, resignation accepted to take effect Oct. 2, 1879. Oct. 2, 1879.

*Brown*, J. A., surgeon, resignation accepted to take effect Nov. 1, 1879. Nov. 1, 1879.

*Dana*, C. L., assistant surgeon, resignation accepted to take effect Nov. 15, 1879. Nov. 11, 1879.

